# Vessel elements of two thelypteroid ferns-part I

**DOI:** 10.1186/s40529-020-0281-y

**Published:** 2020-01-27

**Authors:** Swastika Laskar, Utsha Ghoshal, Kakali Sen

**Affiliations:** Department of Botany, University of Kalyani, Kalyani, Nadia, West Bengal 741235 India

**Keywords:** Vessel elements, SEM imaging, Costa, *Ampelopteris prolifera*, *Thelypteris interrupta*

## Abstract

**Background:**

Hydraulically efficient xylem was evolved in the vascular plants as an apomorphy of the group. Main xylem components involved in water conduction are tracheid and vessel. Vessels, in which two ends are perforated, constituted major evolutionary innovation within vascular plants, presumably providing more efficient solute conduction. Not all vascular plants have vessels. In pteridophytes vessels are present only in seven genera. The contention lies regarding the presence and distribution of vessel in pteridophytes are the impulsive force of this investigation.

**Methods:**

Tracheary elements are isolated following the standard maceration technique, then hand-razor cut longisections are passed through the aqueous alcohol grades and air-dried samples are placed on stub, sputter coated with gold and examined with SEM.

**Results:**

Two thelypteroid ferns viz. *Ampelopteris prolifera* (*Retz.*) Copel. and *Thelypteris interrupta* (Willd.) K. Iwats. are having vessel elements in root, rhizome, stipe, rachis, primary vein/costa, root-rhizome and rhizome-petiole junction i.e. through entire vascular connection of the plant body though the vessel network is interrupted and joined with parenchyma at the end in some places. Presence of vessel elements in the costa of pteridophytic taxa is first time reported by this study. Vessel end-walls are obliquely placed (root, rhizome, and stipe) but oblique to horizontal orientation is noticed in the primary vein/costa. End-walls are with simple, intermediate and compound perforation plates observed through SEM imaging as well as with tissue specific stain. Studied taxa are grown either in terrestrial microclimate of two contrasting environments i.e. sun and shade (*A. prolifera*) or in open swampy land (*T. interrupta*) with moderate to highly disturbed places as rapid proliferating populations showing interpopulation variations of tracheary elements length–width(s) and vessel end-wall length–width(s).

**Conclusion:**

Vessel elements are present throughout the entire vascular connections of the plant body of *A. prolifera* (*Retz.*) Copel. and *T. interrupta* (Willd.) K. Iwats. Interpopulation variation of tracheary elements length–width(s) and vessel end-wall length width(s) are noticed. Till date only seven genera of pteridophytes are reported for the presence of vessel and these two genera are the new addition with the previous.

## Background

Non-vascular plants recover from complete desiccation by poikilohydry, a metabolically expensive phenomenon and with due course of time homoihydry evolved in the vascular land plants with hydraulically efficient xylem would be the major apomorphy (Proctor and Tuba 2002; Niklas 2000; Pittermann et al. 2015). Evolution proceeds in the route of progressive innovations for cheap water transport and evolved in the order with capillary suction at cell walls, stomatal regulations, hydroids, tracheids, secondary xylem, endodermis, vessels (Sperry 2003). An essential evolutionary pressure towards the development of vessel is to reduce the number of wall-crossings and the hydraulic resistance to flow within the xylem (Comstock and Sperry 2000).

Vessels are specially built up of a longitudinal series of individual cells, termed vessel members or elements to provide the three dimensional pathway for the ascent of water in plants, having perforations at both the ends, sometimes in the lateral wall. Other regions of the wall are highly lignified and water moves freely through perforations in the end-walls. Perforation occurs at the last stage of vessel element differentiation as an event of programmed cell death (Esau 1965; Esau and Charvat 1978; Buvat 1989). A differentiating vessel element is connected with a mature dead vessel element and a vessel precursor cell at each end but perforations occur only at the end-wall between the mature dead vessel element and the differentiating vessel element (Esau and Charvat 1978). Vessel elements are present in *Gigantopteris* (Permian fossil seed plants), Gnetales, some extant lycophyte and ferns and in angiosperms except some genera of basal clades (Li et al. 1996; Bailey and Tupper 1918; Carlquist and Schneider 2007; Taylor and Wilson 2012).

Since the first report of vessel in seedless vascular plants by Russow (1872) in *Pteridium aquilinum*, a handful of paper was published and withdrawn due to the imperfect methodology followed by the workers and later on  a revised and well accepted concept was proposed by Carlquist and Schneider (2007), following the recent technique reinvestigations revealed vessel bearing four genera of ferns viz. *Woodsia*, *Marsilea, Astrolepis* and *Pteridium* and one lycophyte i.e. *Selaginella* by Carlquist and Schneider (2007) and two more genera i.e. *Aleuritopteris* and *Cheilanthes* by Sen and Mukhopadhyay (2014). But it may be possible that vessels can present in a great number of species that we currently know (Pittermann et al. 2011).

This is a revisit to explore the distribution of vessels in the pteridophytic taxa following the new concept to remove the contention lies in this regard. Two thelypteroid taxa viz*. Ampelopteris prolifera* and *Thelypteris interrrupta* are selected first as noticed to present as continuous patches on the moist climatic zone of West Bengal and the habitats are facing different levels of disturbance, till the taxa are present as rapidly proliferating populations. The first one prefers terrestrial microclimate and the second one invaded the roadside open swampy land or sometimes found in the barren drier land devoid of water supply. Both the taxa are found to grow extensively in their respective niches and outcompete other associated plant components. Vessel elements and other xylem components (protoxylem and metaxylem tracheids) i.e. the evolutionary antecedents of vessels are also studied, to see the plexus of vascular connections in the different organs of the plant body and the respective distribution of different types of xylary elements.

## Methods

Aim of the present study lies with the investigation of tracheary elements especially to observe the vessels present in the studied taxa and whether any interpopulation variations of tracheary element length–width(s) are present with the microclimatic condition that is sun and shade grown plants facing different level of disturbance (high to moderate level of disturbance) and in undisturbed condition.

**Fig. 1:**
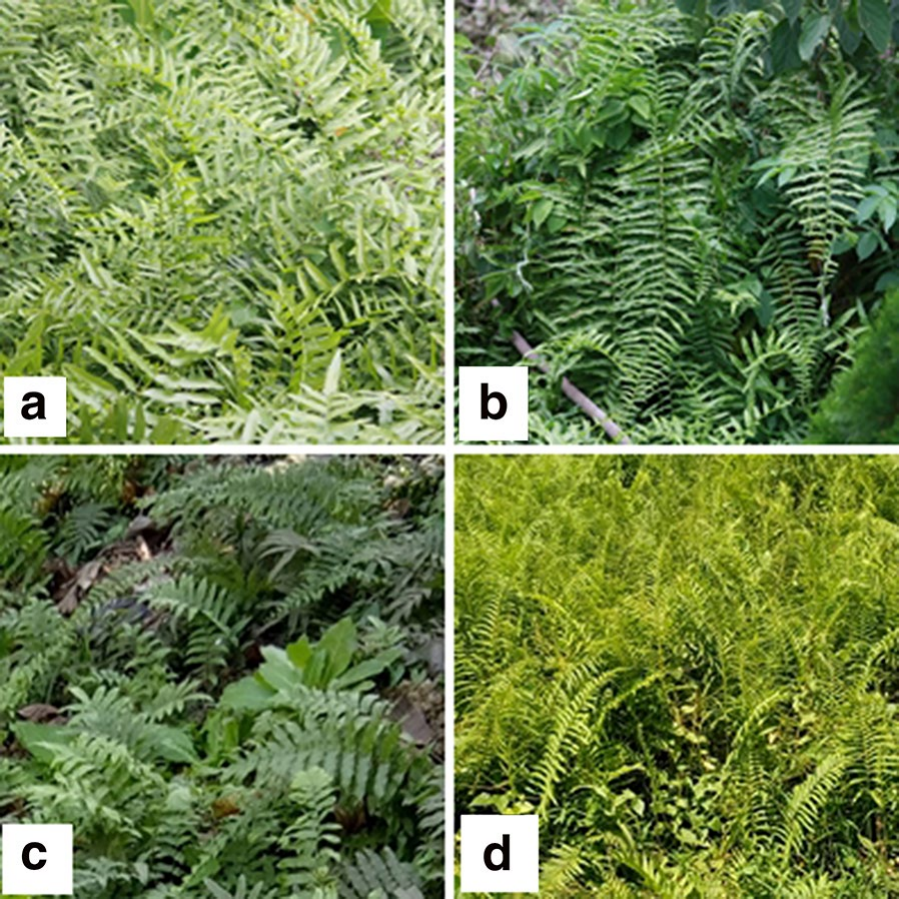
Studied population of **a** *Ampelopteris prolifera* (open environment from University Campus), **b** *A. prolifera* (shade environment from University Campus), **c** *A. prolifera* (semishade population from Chakdah area near river Ganges)** d** *Thelypteris interrupta* (Open swampy environment near Magra–Talandu Railway Track)

Materials were collected from the lower Gangetic plain area of West Bengal from two contrasting environments (if available) of each site. Geographical and climatological data of respective specimen collection sites are mentioned in Table 1. Their distribution is observed throughout the different districts of West Bengal to see their habitat and climatic preference. *Ampelopteris prolifera* were studied from six populations of three places viz. disturbed [continuous human interference on highway side, population 5 open (P3O) and population 6 shade (P3S)], moderately disturbed [infrequent human interference in the Kalyani University campus, population 1 open (P1O), population 2 shade (P1S) and near riverside, Chakdaha population 3 semishade (P2Ss)] and undisturbed [a little bit away from riverside, Chakdaha inside dense canopy where human interference is rare, population 4 shade (P2S)]. Two contrasting microenvironments were selected from each three places that is sun (exposed on bare ground, P1O, P3O) and shade/semi shade (below canopy or at the shade of large buildings, P1S P2Ss, P2S, and P3S) (Fig. 1a, b). *Thelypteris interrupta* were found to grow in similar environments everywhere. Mainly the railway side/roadside wetlands or lowlands were occupied by this plant, of which two populations were selected one from Halisahar, North 24 Pargana (waterlogged wet land, heavy growth of the plant through hectors of area)/P1Sw.O and another from Mogra, Hooghly (near railway side barren dried land surviving with remnants of populations)/P2D.O. (Fig. 1c, d).

**Table 1:** Geographical position and climatological data of sampling sites

Name of the taxa	Sample collection site	Site description	Geographical position	Altitude (m)	Mean annual temperature (minimum) (°C)	Mean annual temperature (maximum) (°C)	Mean annual temperature (average) (°C)	Mean annual rainfall (mm)
*Ampelopteris prolifera*	Kalyani University	Open Shade	22.9751° N, 88.4345° E	11	19.2	30.5	26.3	1345
	Chakdaha	SemiShade Shade	23.0479° N, 88.5130° E	11	*19*	*30*	26.3	*1353*
	Kalyani Expressway	Open Shade	22.9751° N, 88.4345° E	11	19.2	30.5	26.3	1345
*Thelypteris interrupta*	Magra (near Abacus Institute)	Open Beside railway track Dry habitat	23.12205° N, 88.2879° E	16	16	38	26.8	1500
	Halisahar	Open Swampy area	22.932° N, 88.419° E	15	19	45	26.8	1579

**Fig. 2:**
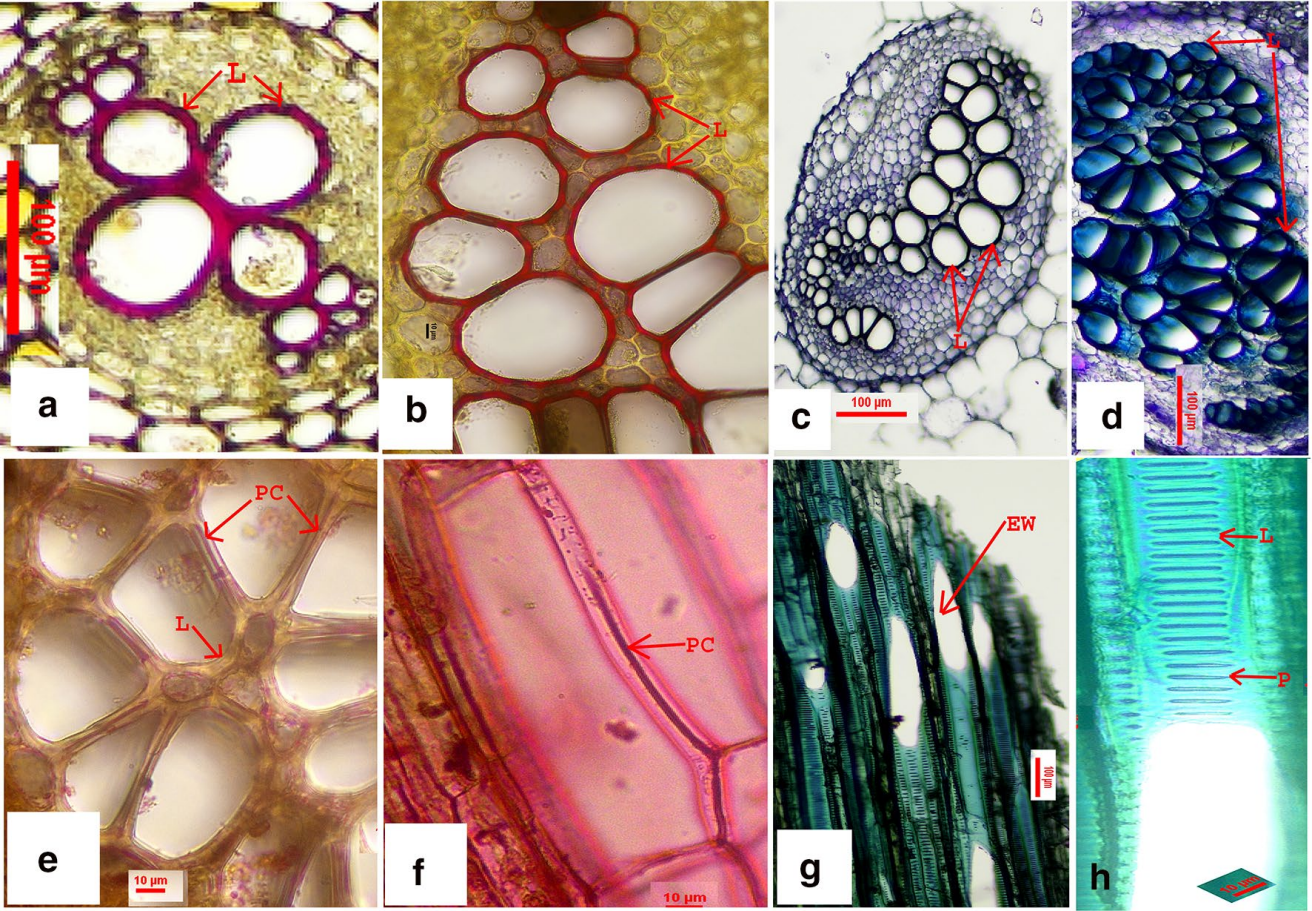
Tracheary elements of *Ampelopteris prolifera*  showing histochemical differentiation in tissue specific stain. **a** Vessel elements in root t.s. **b**–**d** Petiole t.s.(basal segment). **b** Lignified tissue stained with phloroglucinol-HCl. **c**, **d** Lignified tissue stained with toluidine blue o. **e**, **f** Pecto cellulosic middle lamella stained with ruthenium red. **g**, **h** Negatively stained endwall and stained lignified bars in the lateral wall in the l.s. of petiole showing vessel elements. (Abbreviations used: *L* lignin, *PC* pectocellulosic middle lamella, *EW* endwall, *P* perforation)

**Fig. 3:**
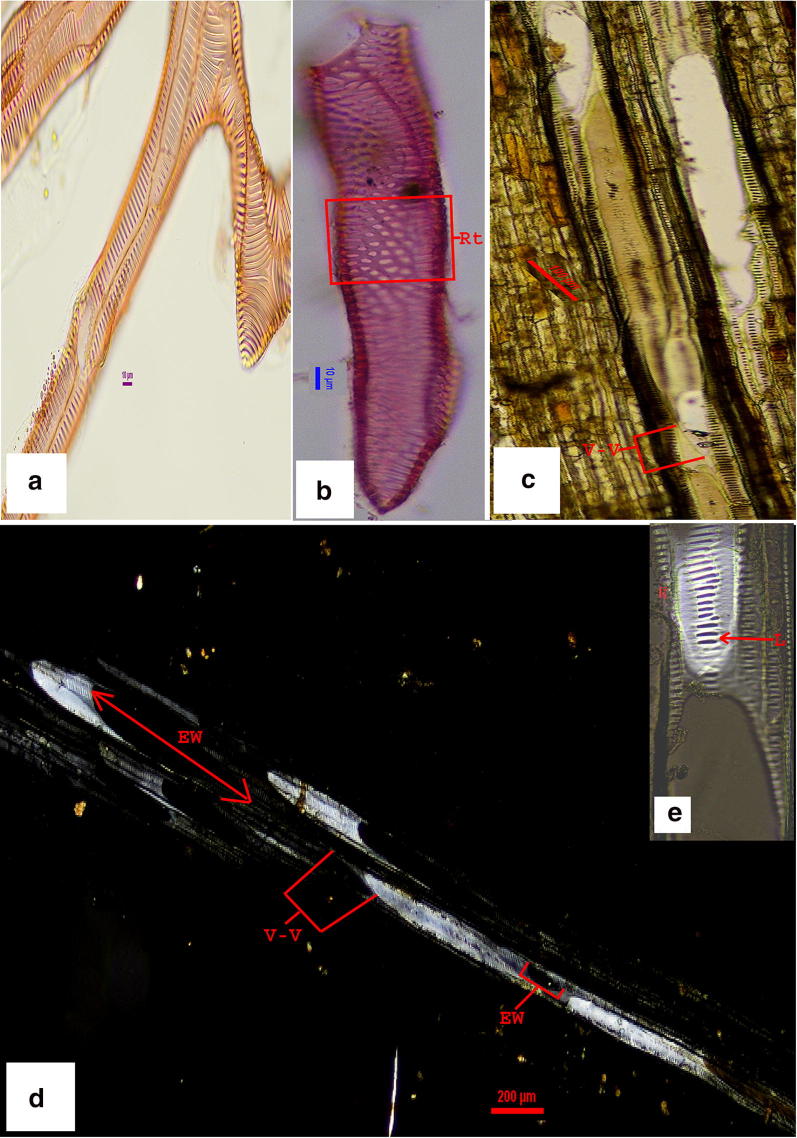
**b** Vessel elements of costa (*Ampelopteris prolifera*) showing reticulate thickening at its lateral wall. **a** Branched vessel elements from macerated root–rhizome junction. **c**–**e** (petiole L.S. of *Thelypteris interrupta*). **c** Vessel–vessel connection of unstained section. **d** Vessel element–vessel element connection in polarized light. **e** Enlarged view of single vessel member—lignified wall glittering in polarized light and the endwall portion empty (blacken part) due to the absence of lignified tissue (Abbreviations used: *Rt* reticulate thickening, *EW* endwall, *V–V* two vessel members are joined end to end)

### Maceration study

For each population tracheary elements of different plants were studied to see the organ wise variations (i.e. in root, rhizome, petiole, lamina, root-rhizome junction, rhizome-petiole junction and costa). First, the tracheary elements were isolated following the standard maceration technique (Johanssen 1940); measurements were taken from the macerated material. Length and width of protoxylem and metaxylem tracheids, vessels, and endplate of the vessel elements. For each of the plant parts slides were prepared from different plants. Minimum 15 readings were taken and mean and standard deviations were calculated in MS Excel. Taxa mean was calculated from population mean for each of the plant organ. The tissue somewhat distal in position from the apical parts and the portion of roots adjacent to the rhizome were taken for study.

### SEM imaging

Hand-razor cut longisections were observed in the light microscope then passed though aqueous alcohol grades and air-dried samples were placed on stub, sputter coated with gold and examined with SEM (model Zeiss EVO-MA 10) (Carlquist and Schneider 2007).

### Histochemical and optical tests

Histochemical and optical tests of vessel end-walls were done by using different tissue specific stains like phloroglucinol–hydrochloric acid and Toludine blue O (lignified wall), ruthenium red (pecto-cellulosic middle lamella), (Johanssen 1940; Yata et al. 1970) and the sections were also observed in polarized light (idea was developed and established by the corresponding author of this manuscript) to see the lignin free zone and imaging in Nikon E200 microscope.

## Results

*Ampelopteris prolifera* (*Retz.*) *Copel*. and *T. interrupta* (Willd.) K. Iwats, both plants grow as a continuous patch in roadside dry/wetlands outcompeting the other plant components of the respective field as well. *A. prolifera* is grown in sun/shade terrestrial microclimate and *T. interrupta* always prefer open land. The plants invaded undisturbed to highly disturbed habitats by rapid proliferation. Their distribution is observed throughout West Bengal and it was seen that the moist climatic part of the state is congenial for their rapid proliferation because these plants are represented very poor or not at all in the drier parts of the state.

### Comparison of interpopulation length–width variations of tracheary elements in the different organ of the studied taxa

Tracheary elements i.e. protoxylem, metaxylem and vessel elements length–width(s) and the vessel end wall length width(s) are presented in Table 2 for *A. prolifera* and Table 3 for *T. interrupta*.

**Table 2:** Tracheary elements and vessel member end wall length–width(s) of *Ampelopteris prolifera* from different population

Habitat	Plant organ	Protoxylem				Metaxylem			
		Length		Width		Length		Width	
		Range	M ± sd	Range	M ± Sd	Range	M ± sd	Range	M ± sd
P1 O	Root	0.50–1.46	0.88 ±0.22	0.008–0.029	*0.016* ± 0.005**	5.32–6.57	5.94 ± 0.32	0.02–0.05	0.042 ± 0.01
	Rhizome	0.43–0.52	0.49 ± 0.02	0.007–0.019	0.013 ± 0.003	–	–	–	–
	Petiole	0.85–1.78	*1.07* ± 0.25**	0.012–0.029	*0.023* ± 0.003**	2.25–4.68	*3.19* ± 0.66*	0.02–0.048	0.036 ± 0.007
	Frond	0.892–1.84	1.19 ± 0.24	0.008–0.035	0.020 ± 0.006	2.17–4.43	3.18 ± 0.59	0.12–0.023	0.020 ± 0.003
P1 S	Root	1.05–2.18	*1.49* ± 0.36**	0.008–0.019	0.013 ± 0.003	5.71–8.00	*6.73* ± 0.63**	0.019–0.070	0.044 ± 0.019
	Rhizome	0.35–0.86	0.60 ± 0.13	0.005–0.024	0.014 ± 0.006	–	–	–	–
	Petiole	0.44–1.17	0.92 ± 0.20	0.007–0.024	0.019 ± 0.005	5.09–7.04	6.27 ± 0.55	0.041–0.078	*0.054* ± 0.009**
P2 Ss	Root	0.81–3.08	1.30 ± 0.74	0.007–0.026	0.015 ± 0.006	4.29–8.28	6.43 ± 1.18	0.023–0.064	0.035 ± 0.011
	Rhizome	0.16–0.23	*0.20* ± 0.02*	0.007–0.019	0.011 ± 0.003	–	–	–	–
	Petiole	0.65–1.11	0.89 ± 0.16	0.008–0.022	0.015 ± 0.004	5.77–11.65	*6.62* ± 1.96**	0.027–0.042	0.035 ± 0.004
P2 S	Root	0.035–1.03	0.62 ± 0.20	0.003–0.019	*0.01* ± 0.004*	1.82–3.24	*2.44* ± 0.047*	0.027–0.038	*0.031* ± 0.003*
	Rhizome	0.51–1.00	0.73 ± 0.018	0.005–0.013	*0.009* ± 0.002*	1.11–2.4	1.9 ± 0.48	0.02–0.028	0.024 ± 0.002
	Petiole	0.38–1.38	0.82 ± 0.28	0.005–0.018	*0.011* ± 0.003*	2.48–3.91	2.86 ± 0.47	0.039–0.045	0.042 ± 0.001
P 3 O	Root	1.06–1.30	1.12 ± 0.063	0.003- 0.018	*0.01* ± 0.004*	3.18–7.7	6.47 ± 1.06	0.029–0.074	*0.06* ± 0.01**
	Rhizome	0.83–1.18	*0.95* ± 0.10**	0.012–0.02	0.014 ± 0.002	–	–	–	–
	Petiole	0.578–0.809	0.70 ± 0.08	0.012–0.024	0.016 ± 0.004	3.06–3.78	3.42 ± 0.026	0.019–0.039	0.030 ± 0.006
P 3 S	Root	0.48–0.74	*0.60* ± 0.06*	0.007–0.018	0.013 ± 0.003	2.95–3.68	3.11 ± 0.21	0.040–0.069	0.048 ± 0.007
	Rhizome	0.32–0.38	0.37 ± 0.014	0.021–0.036	*0.028* ± 0.004**	–	–	–	–
	Petiole	0.35–0.95	*0.53* ± 0.19*	0.014–0.027	0.020 ± 0.003	2.85–3.95	3.40 ± 0.37	0.016–0.055	0.040 ± 0.011

Habitat	Plant organ	Vessel element				Vessel element Endplate			
		Length		Width		Length		Width	
		Range	M ± sd	Range	M ± sd	Range	M ± sd	Range	M ± sd
P1 O	Root	1.50–2.77	2.25 ± 0.47	0.019–0.038	*0.03* ± 0.004*	103.9–315.05	205.28 ± 54.3	10.92–34.67	21.67 ± 5.90
	Rhizome	0.78–2.4	1.59 ± 0.43	0.01–0.069	0.04 ± 0.01	127.3–527.02	249.96 ± 94.15	12.18–29.02	23.04 ± 5.41
	Petiole	3.05–8.84	5.62 ± 1.59	0.039–0.055	0.04 ± 0.004	243.6–939.99	*584.47* ± 196.60**	10.90–38.25	30.1 ± 16.13
	Frond	1.78–3.31	2.31 ± 0.47	0.017–0.032	0.026 ± 0.004	119.6–543.2	281.65 ± 99.76	5.45–27.14	17.18 ± 5.91
P1 S	Root	0.87–5.10	*2.16* ± 0.92*	0.033–0.057	0.043 ± 0.008	210.1–689.16	390.56 ± 106.24	13.46–24.36	21.44 ± 4.37
	Rhizome	0.99–1.57	*1.19* ± 0.16*	0.026–0.052	0.039 ± 0.007	41.67–682.02	*340.53* ± 176.09**	17.23–39.29	*30.49* ± 6.27**
	Petiole	1.05–2.82	*2.21* ± 0.46*	0.008–0.022	*0.018* ± 0.003*	124.2–219.04	*173.89* ± 25.7*	24.36–35.52	30.93 ± 3.19
P2 Ss	Root	2.43–4.89	3.55 ± 0.69	0.023–0.038	0.034 ± 0.004	345.2–484.86	*421.34* ± 40.09**	7.70–47.65	25.31 ± 12.8
	Rhizome	0.74–2.45	1.55 ± 0.046	0.029–0.067	0.044 ± 0.012	23.11–130.98	*65.24* ± 32.96*	3.85–34.67	18.21 ± 8.33
	Petiole	0.82–7.02	4.11 ± 1.64	0.023–0.081	0.041 ± 0.018	99.41–492.56	290.7 ± 138.46	11.56–37.15	24.60 ± 9.01
P2 S	Root	1.23–4.02	2.78 ± 0.76	0.023–0.05	0.039 ± 0.007	79.32–675.62	250.73 ± 161.05	15.88–31.06	*24.14* ± 4.69**
	Rhizome	0.60–3.09	1.87 ± 0.54	0.022–0.055	*0.038* ± 0.009*	31.77–307.04	173.49 ± 73.88	8.31–26.27	19.39 ± 5.46
	Petiole	0.94–6.92	4.27 ± 1.83	0.033–0.11	*0.064* ± 0.02**	373.7–935.46	534.81 ± 149.7	24.36–36.34	31.55 ± 2.83
P 3 O	Root	0.62–4.28	2.99 ± 1.09	0.028–0.066	0.044 ± 0.009	33.66–709.97	308.52 ± 232.2	5.25–26.28	*14.56* ± 6.48*
	Rhizome	1.47–4.21	*2.22* ± 1.05**	0.029–0.071	0.053 ± 0.012	33.31–329.27	92.25 ± 76.54	3.85–63.77	20.74 ± 15.10
	Petiole	4.2–6.73	5.02 ± 0.71	0.028–0.052	0.038 ± 0.009	83.34–662.41	315.46 ± 171.17	7.7–24.67	*15.3* ± 5.06*
P 3 S	Root	4.95–11.45	*7.32* ± 1.55**	0.031–0.078	*0.053* ± 0.015**	32.69–322.53	*109.57* ± 84.15*	11.51–34.88	20.76 ± 6.54
	Rhizome	1.06–3.49	2.08 ± 0.72	0.034–0.072	*0.054* ± 0.013**	42.55–382.75	164.50 ± 123.85	5.45–31.06	*17.21* ± 8.73*
	Petiole	3.95–9.76	*7.37* ± 1.80**	0.023–0.080	0.045 ± 0.016	112.3–1097.8	487.70 ± 267.16	19.26–41.67	*33.09* ± 7.11**

In each case range (min.–max.) and mean ± SD value is given. Measurement unit of tracheary elements length–width is in millimetre and vessel end wall is in micrometre. Italic font with double superscript shows longest and italic font with single superscript shows shortest tracheary elements of different plant parts of  the studied population

*P1O* Population 1 open, *P1S* population 2 shade (Kalyani University Campus), *P2Ss* population 3 semishade, *P2S* population 4 Shade (Chakdaha); *P3O* population 5 Open, *P3S* population 6 shade (Highway side)

**Table 3:** Tracheary elements and vessel member end wall length–width(s) of *Thelypteris interrupta* from different population

Habitat	Plant organ	Protoxylem				Metaxylem			
		Length		Width		Length		Width	
		Range	M ± sd	Range	M ± sd	Range	M ± sd	Range	M ± sd
P1 Sw.O	Root	0.668–1.46	*1.29* ± 0.023*	0.007–0.016	*0.012* ± 0.003**	2.014–7.602	*5.780* ± 0.154*	0.03–0.076	*0.056* ± 0.015**
	Rhizome	0.405–0.069	*0.564* ± 0.096*	0.005–0.028	*0.017* ± 0.006**	2.564–0.012	*2.564* ± 0.057**	0.027–0.069	*0.048* ± 0.012**
	Petiole	0.742–1.472	*1.071* ± 0.023*	0.008–0.019	*0.016* ± 0.003**	4.325–7.114	*5.380* ± 0.101*	0.023–0.059	*0.041* ± 0.012*
	Frond	0.436–1.40	0.885 ± 0.22	0.017–0.029	0.021 ± 0.003	3.80–6.25	4.51 ± 0.64	0.027–0.04	0.032 ± 0.004
P2 D. O	Root	0.439–2.866	*1.48* ± 0.005**	0.005–0.022	*0.011* ± 0.066*	6.62–9.91	*7.79* ± 0.113**	0.024–0.064	*0.051* ± 0.095*
	Rhizome	0.28–0.71	*0.59* ± 0.01**	0.005–0.019	*0.013* ± 0.005*	1.481–2.542	*2.33* ± 0.014*	0.012–0.038	*0.019* ± 0.011*
	Petiole	0.513–2.109	*1.45* ± 0.058**	0.005–0.022	*0.011* ± 0.006*	4.560–8.858	*6.306* ± 0.131**	0.025–0.071	*0.054* ± 0.013**

Habitat	Plant organ	Vessel element				Vessel element Endplate			
		Length		Width		Length		Width	
		Range	M ± sd	Range	M ± sd	Range	M ± sd	Range	M ± sd
P1 Sw.O	Root	1.590–6.145	*4.158* ± 0.102*	0.021–0.095	*0.06* ± 0.025**	212.15–442.26	*306.98* ± 75.56**	20.01–39.33	*29.67* ± 7.25**
	Rhizome	1.284–1.720	*1.529* ± 0.18**	0.041–0.115	*0.076* ± 0.02**	197.3–461.77	*292.42* ± 58.63**	28.04–54.07	*44.01* ± 11.06**
	Petiole	1.866–5.197	*3.006* ± 0.12**	0.018–0.065	*0.032* ± 0.013*	357.5–987.94	*640.08* ± 20.28**	29.93–69.31	*45.83* ± 13.40*
	Frond	1.90–2.56	2.24 ± 0.18	0.019–0.032	0.024 ± 0.004	171.07–481.34	328.55 ± 73.41	15.88–28.04	21.51 ± 3.12
P2 D. O	Root	7.271–4.209	*5.131* ± 0.11**	0.053–0.031	*0.045* ± 0.008*	62.12–465.23	*101.01* ± 58.89*	17.23–22.46	*19.22* ± 1.93*
	Rhizome	3.250–1.00	*1.325* ± 0.015*	0.064–0.024	*0.040* ± 0.012*	103.3–348.26	*205.77* ± 70.88*	5.45–15.41	*13.39* ± 3.39*
	Petiole	2.775–1.639	*2.363* ± 0.098*	0.067–0.052	*0.057* ± 0.01**	73.6–356.39	*175.7* ± 96.54*	15.41–56.21	*46.70* ± 14.13**

In each case range (min.–max.) and M (mean) ± SD value is given. Measurement unit of tracheary elements length–width is in millimetre and vessel end wall is in micrometre. Italic font with double superscript denotes longest tracheid length and italic font with single superscript denotes shortest tracheid length of the respective organ from the studied population

*P1Swo* Population 1 swampy open land; *P2DO* population 2 of drier open

In the Tables 2, 3 longest and shortest tracheary elements of different organ are shown with bold font double and single superscript respectively by considering the population level variation. Among all three types of tracheary elements in root metaxylem components are longer and wider in all the population of *A. prolifera* except in population 6 i.e. P3S where vessel elements are longer than the metaxylem components. In *T. interrupta* also root metaxylem components are longer and wider but in population 1 vessel elements are wider than the metaxylem components. In rhizome of *A. prolifera* the vessel elements and protoxylem tracheids are found and no metaxylem components are found except in population 4 i.e. P2S whereas in *T. interrupta* metaxylem components are longer than the other two elements and vessel elements are wider than the metaxylem. In petiole of *A. prolifera* vessel elements are longer in four populations i.e. P1O, P3O, P2S, P3S and metaxylem components are longer than the vessel elements in rest two populations i.e. P1S and P2Ss. Metaxylem components are wider than the vessel elements in P1S and in rest five populations’ vessel elements are wider. In *T. interrupta* petiole metaxylem components are longer than the vessel elements. In the costa of *A. prolifera* metaxylem components are longer and vessel elements are wider. In *T. interrupta* costa metaxylem components are longer and wider than the vessel elements.

In *A. prolifera* longest protoxylem tracheid i.e. 1.49 ± 0.36 mm is observed in the root of P1S and shortest protoxylem tracheid length is observed in the rhizome of P2Ss i.e. 0.20 ± 0.02 mm. Metaxylem tracheids of rhizome are absent in all the populations except one that is in population 4 (P2S). Longest metaxylem tracheid i.e. 6.73 ± 0.63 mm was observed in the root of population 2 (P1S) and shortest i.e. 2.44 ± 0.047 mm in the root of population 4 (P2S). Longest vessel element was seen in the root of P3S i.e. 7.37 ± 1.80 mm and shortest in the rhizome i.e. 1.19 ± 0.16 mm. Longest vessel element endplates are seen in petiole of P1O i.e. 584.40 ± 196.60 µm in *A. prolifera* and shortest in rhizome of P2Ss i.e. 65.24 ± 32.9 µm.

In *T. interrupta* longest protoxylem tracheid was found in the root of population 2 i.e. 1.479 ± 0.005 mm and shortest in the rhizome of population 1 i.e. 0.564 ± 0.096. Metaxylem tracheid was longest in root of P2DO i.e. 7.79 ± 0.113 mm and shortest in the rhizome of P2DO i.e. 2.33 ± 0.014 mm. Root vessel element of P2 DO was longest i.e. 5.13 ± 0.105 mm and shortest in the rhizome of P2DO i.e. 1.325 ± 0.015 mm. In *T. interrupta* longest vessel element endplate was seen in petiole of P1SWO i.e. 640.08 ± 20.28 µm and shortest in root of P2DO 101.01 ± 58.89 µm.

In both the taxa protoxylem tracheids are with spiral thickening and metaxylem tracheids are with scalariform thickening of opposite and alternate patterns. End-walls are pointed or tapered. Branched tracheids are observed in lamina/costa and rhizome of both the taxa.

### Comparison of mean length–width variation of tracheary elements in the studied taxa

In Table 4 a comparison of mean length–width of two taxa is provided. Taxa mean was calculated from population mean of each organ. Among vessel elements of all the organ petiole vessel elements are longer in *A. prolifera* i.e. 4.77 mm and root vessel elements are longer in *T. interrupta* i.e. 4.64 mm. Width is similar of all the organ in case of vessel elements of *A. prolifera* i.e. 0.04 mm and among all the plant parts in *T. interrupta* rhizome vessels are wider i.e. 0.06 mm.

**Table 4:** Comparison of tracheary elements length–width(s) of the two studied taxa (measurement unit mm)

Plant part	Tracheary element	*Ampelopteris prolifera*		*Thelypteris interrupta*	
		Mean length	Mean width	Mean length	Mean width
Root	Protoxylem	1.00	0.01	1.38	0.01
	Metaxylem	5.19	0.04	6.79	0.05
	Vessel	3.51	0.04	4.64	0.05
Rhizome	Protoxylem	0.56	0.01	0.58	0.02
	Metaxylem	1.9	0.02	2.45	0.03
	Vessel	1.75	0.04	1.43	0.06
Petiole	Protoxylem	0.82	0.02	1.26	0.01
	Metaxylem	4.29	0.04	5.84	0.05
	Vessel	4.77	0.04	2.62	0.03

Taxa mean was calculated from population mean for each organ

In comparison of two studied taxa tracheary elements of all the types are longer in the different organ of *T. interrupta* than the first one but only the vessel elements are longer in the rhizome of *A. prolifera* than the second taxa but wider in *T. interrupta* than the first one. The petiole vessel elements of *A. prolifera* are wider than the second one.

### Organographic distribution of vessel elements in the studied taxa

**Fig. 4:**
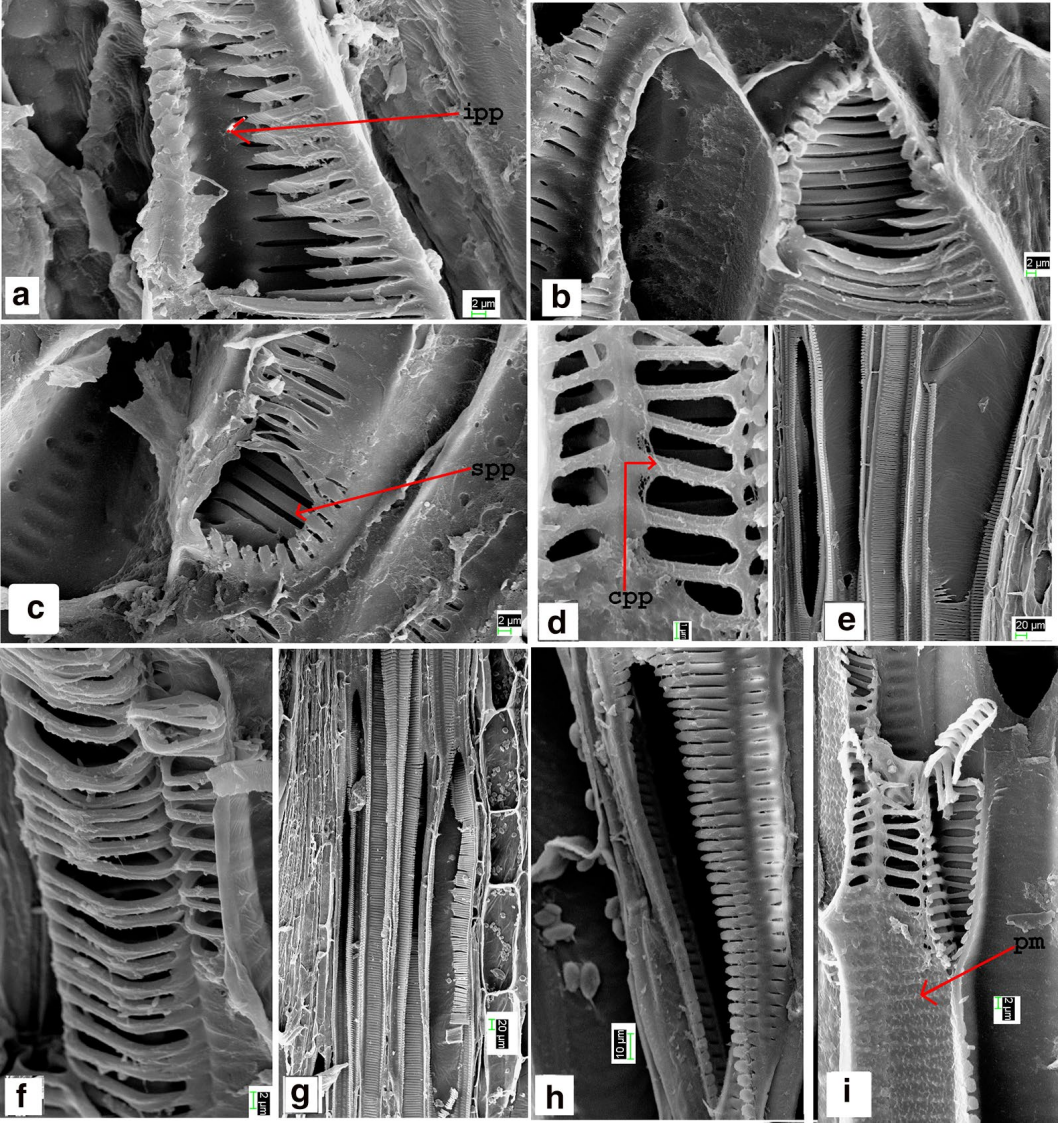
SEM images of *Ampelopteris prolifera*-vessel elements. **a**–**c** Vessel end wall of rhizome **a** with intermediate type of perforation plate, scalariform bars half produced **b** with 2–3 remnant scalariform bars. **c** Simple perforation plate, **d** vessel endwall (root) with complex perforation plate. **e** Vessel element (rhizome) with elongated perforation plate. **f** Helically thickened tracheid. **g** Vessel element with enlarged endwall and intermediate perforation plate associated with xylem parenchyma. **h** Vessel endwall with simple perforation plate. **i** Endwall with compound perforation plate and lateral wall with pit membrane. (Abbreviations used: *ipp* intermediate perforation plate, *spp* simple perforation plate, *cpp* compound perforation plate, *pm* pit membrane)

Vessels are found in all the plant parts including midrib/costa of the lamina in both cases. Vessels are with inclined and horizontal endplate. Endplates are with simple (Fig. 4c, h), compound (Fig. 4d) or intermediate type of perforation plate (Fig. 4a, b). In the present observation vessel elements are found to be present in all the plant parts (that is in root, rhizome, petiole, rachis and in the primary vein of the pinnae) of the genera. The uniqueness of the endplate is having completely (Fig. 4d) or incompletely formed (Fig. 4a, b, g) scalariform bar or simple perforation plate (Fig. 4c, h, e) at the end-wall. The end-walls are always inclined/obliquely placed except in the midvein of *A. prolifera* (Fig. 3b). In the vessel elements of primary vein the secondary thickening of the lateral wall is of reticulate type (Fig. 3b) and the end-wall tends to be horizontal. In the root-rhizome junction (Fig. 3a) and the rhizome petiole junction vessel elements are present.

### Histochemical and optical tests of endwalls of vessel elements

Lignified tissues of phloroglucinol–hydrochloric acid stained sections are showing reddish (Fig. 2a, b) and vessel end-walls show negative reactions. Transections of root and petiole showing vessel elements occupy the central part of the stele stained positively with phloroglucinol–hydrochloric acid (Fig. 2a, b) and toludine blue O (Fig. 2c, d). Toludine blue O which stains lignin blue did not stain the end-wall (Fig. 2g, h). Walls with pectic substances are stained red with ruthenium red (Fig. 2e, f). Very week double refraction in polarized light from the end-wall indicates their cellulosic nature and lignified part is refracted when seen in cross polarized light (Fig. 3d, e).

### Connections of vessel elements and associations

Vessels are associated either with xylem parenchyma in both the sides or with metaxylem and protoxylem tracheids in the other end (Fig. 3c). Vessel members are joined end to end and form vessel network (Fig. 3c, d). Vessel–vessel inter connections and the interruption of the vessel network was observed from a part of tissue in longitudinal sections in white light (Fig. 3c) and cross polarized light (Fig. 3d, e).

## Discussion

Over 60 species of pteridophytes have managed to invade both intact and disturbed ecosystems, often outcompeting and even smothering native angiosperms and conifers (Robinson 2010). The reasons behind the ferns’ competitive edge are complex but a physiological approach may help explain their rapid rates of spread, as well as the mechanism by which these species push their xylem function and overall physiology beyond the norm. Studied two taxa were also of similar nature always outcompeting the associated components and occupy a continuous large area.

Vessel elements are specialized gradually by shifting scalariform to simple perforation plates on their end wall and rotation of highly oblique to transverse/horizontal orientation of the end wall for efficient conduction of water (Bailey and Tupper 1918). Appearance of vessel characters in distantly related plant group is the result of parallel evolution (Baas and Wheeler 1996). Phylogenetic distribution of vessel across vascular plants is: gigantopterids-the Permian seed plants (only fossil representative of gymnosperm bearing vessel), some extant ferns and lycophyte, extant taxa of gnetales and angiosperms except some genera of basal clades. The distribution of the vessel element is not apodeictic in seedless vascular plants. Recent method of vessel detection was established by Carlquist and Schneider (2007); following that, vessel elements are found to be present in the root, rhizome and petiole of *Marsilea* (aquatic), *Selaginella*, *Pteridium aquilinum* (terrestrial), *Astrolepis, Woodsia, Aleuritopteris* and *Cheilanthes* (xeric) (Carlquist and Schneider 2007; Sen and Mukhopadhyay 2014) i.e. total seven genera of pteridophytes bearing vessel belonging to different habitat.

Present investigation reports vessel elements in *A. prolifera* and *T. interrupta* belonging to mesic terrestrial open/shade/semishade and open swampy habitat respectively. Previously reported genera bear vessel elements in root, rhizome and/or petiole but the presently reported genera bear vessel elements in the costa, root-rhizome and rhizome-petiole junction in addition which was never surveyed in any genera of pteridophyte. Presence of vessel elements through entire vascular connections of the studied taxa is established first time by the present study. In monocot secondary xylem vessels are present in the root and absent in leaves and stems later on vessel replaced tracheid in leaf and stem vasculature (Cheadle 1942). Another important feature noticed in *A. prolifera* is that the vessel element end-wall tends to be horizontal in costa and the lateral wall thickening is of reticulate type i.e. rare in pteridophytes. The orientation of endplate gradually changed from root to leaf vasculature, more advanced type is found in root and gradually become primitive to leaf previously mentioned by Cheadle (1942, 1943) is not supported by this study.

All plant organ studied separately, revealed the presence of more elongated end-walls in the petiole. In *T. interrupta* 640.08 ± 20.28 µm longest and in *A. prolifera* 584.40 ± 196.60 µm longest endplate in the petiole were found and the endwalls were with all type of perforation plate i.e. simple (no scalariform bars), intermediate (scalariform bars half formed) and compound (with scalariform bars) type. Series of intermediate characters i.e. compound type of perforation plate, and incompletely formed scalariform bars in the end-wall and simple perforation plates are seen in the vessel end-wall of same plant organ indicating the sequential stages of end wall formation by end wall digestion.

Comparative data of the two taxa show variation of length–width in the different types of tracheary elements (Tables 2, 3 and 4) but shortening of length is not distinctly correlated with gradual increase of width or vice versa in different taxa or the different organs of the same taxa.

In *A. prolifera* five population (except P2S) bear protoxylem and vessel elements in the rhizome but no metaxylem is present. In the aforementioned five populations (P1O, P1S, P2Ss, P3O, P3S) water table is far from the ground and also disturbance (high to moderate) is present. But in the population 4 (P2S) plants where all type of tracheary elements is present were grown near the water and the situation was undisturbed. Sen et al. (1989) reported that vessel formation is regulated by disturbance and different type of stress but no experimental proof was provided. The exact factors controlling the nature and amount of vascular tissue formation need further evidence or experimental data in controlled condition.

Study of the vessel size revealed that the root vessels are longer in *T. interrupta* whereas in *A. prolifera* petiole vessels are more elongated. So the vessel elements are longer in either root or petiole followed by rhizome. *A. prolifera* growing in open habitat are not always showing shorter tracheary elements than the taxa grown in shade habitat (Tables 2, 3) which is present in case of *Aleuritopteris* and *Cheilanthes* (Sen and Mukhopadhyay 2014).

Independent evolution of vessel was taking place in different plant groups as well as within the same plant group evidenced by previous report of vessel member distribution, genera bearing vessel elements belonging to lycophyte (*Selaginella*), core leptosporangiate (*Marsilea*), polypod (*Pteridium*, *Astrolepis, Cheilanthes*, *Aleuritopteris*) and eupolypod II (*Woodsia*; and *A. prolifera*, *T. interrupta* of present report) (Schuettpelz and Pryer 2008; Taylor and Wilson 2012). The presently reported genera belong to eupolypod II. The genera bear vessel elements in seedless vascular plants are widely distributed among distantly related families of pteridophytes which are of recent origin. Till date no extinct members of pteridophytes are reported for the presence of vessel elements. The first extinct plants reported for the presence of vessel elements are Gigantopterids, a seed plant of Permian origin. Later on the character was not continued in all the predecessors of different group of seed plants since Permian, evidenced by some vesselless genera of angiosperm basal clade of early cretaceous origin and of some extant genera of angiosperm basal clade (Taylor and Wilson 2012). Some other anatomical characters like secondary xylem of heterosporous lycopods (Lepidodendron) and bifacial cambium of fossil genus *Sphenophyllum* (only non-seed plants) of Carboniferous and mid-Devonian origin respectively become disappear with due course of time and reappear later in other groups (seed plants) of plant. Appearance and disappearance of vessel throughout the different plant group tell the similar tale. Dearth of knowledge exists regarding the distribution and evolution of vessel elements through the different plant groups, need more evidence from extinct and extant taxa to depict the proper sequence and radiation of vessel character that was happened in nature and become evolutionary boon to their predecessors.

## Conclusion

Vessel elements are present throughout the entire vascular connections of the plant body (i.e. in root, rhizome, stipe, rachis, primary vein/costa, root-rhizome and rhizome-petiole junctions) of *A. prolifera* (*Retz.*) Copel. and *T. interrupta*(Willd.) K. Iwats. Presence of vessel elements in the costa of pteridophytic taxa is first time reported by this study. Interpopulation variation of tracheary elements length–width(s) and vessel end-wall length width(s) are noticed, which do not corroborate the fact that the studied plants of moist shady habitat always possess longer tracheary elements that the population of open habitat. Till date only seven genera of pteridophytes are reported for the presence of vessels and these two genera are new addition with the previous report.

## Data Availability

Data would be available on request to corresponding author.

## Abbreviations

P: population
O: open
S: shade
Ss: semishade
SwO: swampy open
DO: dry open
P1O: population 1 open (*Ampelopteris prolifera* populations)
P1S: population 2 shade (*Ampelopteris prolifera* populations)
P2Ss: population 3 semishade (*Ampelopteris prolifera* populations)
P2S: population 4 shade (*Ampelopteris prolifera* populations)
P3O: population 5 open (*Ampelopteris prolifera* populations)
P3S: population 6 shade (*Ampelopteris prolifera* populations)
P1Sw.O: population 1 swampy open (*Thelypteris interrupta* populations)
P2D.O: population 2 dry open (*Thelypteris interrupta* populations)
SEM: scanning electron microscopy
